# Hedgehog Signaling Inhibition by Smoothened Antagonist BMS-833923 Reduces Osteoblast Differentiation and Ectopic Bone Formation of Human Skeletal (Mesenchymal) Stem Cells

**DOI:** 10.1155/2019/3435901

**Published:** 2019-11-21

**Authors:** Nihal AlMuraikhi, Nuha Almasoud, Sarah Binhamdan, Ghaydaa Younis, Dalia Ali, Muthurangan Manikandan, Radhakrishnan Vishnubalaji, Muhammad Atteya, Abdulaziz Siyal, Musaad Alfayez, Abdullah Aldahmash, Moustapha Kassem, Nehad M. Alajez

**Affiliations:** ^1^Stem Cell Unit, Department of Anatomy, College of Medicine, King Saud University, Riyadh 11461, Saudi Arabia; ^2^Molecular Endocrinology Unit (KMEB), Department of Endocrinology, University Hospital of Odense and University of Southern Denmark, Odense, Denmark; ^3^Cancer Research Center, Qatar Biomedical Research Institute (QBRI), Hamad Bin Khalifa University (HBKU), Qatar Foundation (QF), PO Box 34110, Doha, Qatar; ^4^Histology Department, Faculty of Medicine, Cairo University, Cairo, Egypt; ^5^Department of Cellular and Molecular Medicine, Danish Stem Cell Center (DanStem), University of Copenhagen, 2200 Copenhagen, Denmark

## Abstract

**Background:**

Hedgehog (Hh) signaling is essential for osteoblast differentiation of mesenchymal progenitors during endochondral bone formation. However, the critical role of Hh signaling during adult bone remodeling remains to be elucidated.

**Methods:**

A Smoothened (SMO) antagonist/Hedgehog inhibitor, BMS-833923, identified during a functional screening of a stem cell signaling small molecule library, was investigated for its effects on the osteoblast differentiation of human skeletal (mesenchymal) stem cells (hMSC). Alkaline phosphatase (ALP) activity and Alizarin red staining were employed as markers for osteoblast differentiation and in vitro mineralization capacity, respectively. Global gene expression profiling was performed using the Agilent® microarray platform. Effects on in vivo ectopic bone formation were assessed by implanting hMSC mixed with hydroxyapatite-tricalcium phosphate granules subcutaneously in 8-week-old female nude mice, and the amount of bone formed was assessed using quantitative histology.

**Results:**

BMS-833923, a SMO antagonist/Hedgehog inhibitor, exhibited significant inhibitory effects on osteoblast differentiation of hMSCs reflected by decreased ALP activity, in vitro mineralization, and downregulation of osteoblast-related gene expression. Similarly, we observed decreased in vivo ectopic bone formation. Global gene expression profiling of BMS-833923-treated compared to vehicle-treated control cells, identified 348 upregulated and 540 downregulated genes with significant effects on multiple signaling pathways, including GPCR, endochondral ossification, RANK-RANKL, insulin, TNF alpha, IL6, and inflammatory response. Further bioinformatic analysis employing Ingenuity Pathway Analysis revealed significant enrichment in BMS-833923-treated cells for a number of functional categories and networks involved in connective and skeletal tissue development and disorders, e.g., NF*κ*B and STAT signaling.

**Conclusions:**

We identified SMO/Hedgehog antagonist (BMS-833923) as a powerful inhibitor of osteoblastic differentiation of hMSC that may be useful as a therapeutic option for treating conditions associated with high heterotopic bone formation and mineralization.

## 1. Introduction

The Hedgehog (Hh) signaling pathway plays many important roles during development and postnatal tissue homeostasis including bone formation [1]. In mammals, the hedgehog family is composed of three protein members: Sonic Hedgehog (Shh), Indian Hedgehog (Ihh), and Desert Hedgehog (Dhh) [2, 3]. The negative and positive cellular regulation of Hh is controlled by two transmembrane proteins, Patched (Ptc), a negative regulator of Hh signaling, and Smoothened (Smo), a GPCR-like 7-transmembrane receptor that stimulates downstream signaling in response to Hh [3, 4]. Thus, an inhibition of Hh signaling consequently inhibits SMO activity [3].

Human skeletal (mesenchymal) stem cells (hMSC) are multipotent stem cells found in the bone marrow and can give rise to various mesoderm cell types including bone-forming osteoblasts [5]. The osteoblastic differentiation of hMSCs is controlled by multiple signaling pathways [6] including Hh signaling [1], TGF*β* [7], bone morphogenetic proteins [8], and Wnt/*β*-catenin [9]. The Hh protein enhances the differentiation of hMSCs into osteoblastic lineage and increases the expression of ALP and in vitro formation of the mineralized matrix which are markers of mature osteoblast functions [10]. Moreover, a disruption of the Hh pathway has been associated with a number of developmental bone diseases [1].

Small molecule inhibitors are currently used as valuable tools to decipher the molecular mechanisms regulating stem cell differentiation, and they have potential therapeutic use [11, 12]. Specifically, the small molecule agonist or antagonist regulators of the Hh pathway, could potentially be used to treat bone diseases. Here, we identified a small molecule BMS-833923, an inhibitor of SMO and the Hh pathway, through a small molecule library screen [11] and characterized its functions in vitro and in vivo on hMSC differentiation to bone-forming osteoblastic cells.

## 2. Materials and Methods

### 2.1. Cell Culture

A hMSC-TERT cell line was used in this study as a model for hMSCs [13, 14]. hMSC-TERT expresses all features of primary hMSCs including multipotency and molecular signature [13, 14]. The cells were cultured in Dulbecco's modified Eagle's medium (DMEM), a basal medium supplemented with 4 mM L-glutamine, 4,500 mg/l D-glucose, and 110 mg/l 10% sodium pyruvate, in addition to 10% fetal bovine serum (FBS), 1% penicillin-streptomycin, and 1% nonessential amino acids. All reagents were purchased from Thermo Fisher Scientific Life Sciences, Waltham, MA (http://www.thermofisher.com). Cells were incubated in 5% CO_2_ incubators at 37°C and 95% humidity.

### 2.2. Osteoblast Differentiation

The cells were maintained until 80%–90% confluency was achieved; then, the medium was replaced with osteoblast induction medium (DMEM containing 10% FBS, 1% penicillin-streptomycin, 50 mg/mL L-ascorbic acid (Wako Chemicals GmbH, Neuss, Germany,http://www.wako-chemicals. de/), 10 mM b-glycerophosphate (Sigma-Aldrich), 10 nM calcitriol (1a,25-dihydroxyvitamin D3, Sigma-Aldrich), and 10 nM dexamethasone (Sigma-Aldrich). The stem cell signaling small molecule inhibitor library and BMS-833923 were obtained from Selleckchem Inc. (Houston, TX, http://www.selleckchem.com). Each small molecule inhibitor was added at 3 *μ*M concentration to the osteoblast induction medium, and cells were exposed to the inhibitor throughout the differentiation period. Control cells were treated with osteoblast induction medium containing dimethyl sulfoxide (DMSO) as vehicle.

### 2.3. Cell Viability Assay

Cell viability assay was performed using an alamarBlue assay according to the manufacturer's recommendations (Thermo Fisher Scientific). Cells were cultured in 96-well plates in 300 *μ*L of the medium. On day 10, 30 *μ*L/well of alamarBlue substrate was added (10%) and plates were incubated for 1 hr in the dark at 37°C. Readings were obtained using the BioTek Synergy 2 Microplate Reader (BioTek Instruments Inc., Winooski, VT, US) at fluorescent mode (ex: 530 nm/em: 590 nm).

### 2.4. Quantification of Alkaline Phosphatase Activity

Alkaline phosphatase (ALP) activity quantification was assessed using the BioVision ALP Activity Colorimetric Assay Kit (BioVision, Inc., Milpitas, CA, http://http://www.biovision.com/) with some modifications. The cells were cultured in 96-well plates. At day 10 of osteoblast differentiation, cells were rinsed once with PBS and fixed using 3.7% formaldehyde in 90% ethanol for 30 seconds at room temperature. The fixative was removed and 50 *μ*L/well of p-nitrophenyl phosphate solution was added and incubated for 30–60 minutes. Optical densities were then measured at 405 nm using a SpectraMax/M5 fluorescence spectrophotometer plate reader, and ALP enzymatic activity was then normalized to cell number.

### 2.5. Alkaline Phosphatase Staining

Cells were cultured in a 6-well plate in osteoblast differentiation medium. On day 10, the cells were washed in PBS and fixed in 10 mM acetone/citrate buffer at pH 4.2 for 5 min at room temperature. The fixative was removed and Naphthol/Fast Red stain (0.2 mg/mL Naphthol AS-TR phosphate substrate (Sigma-Aldrich); 0.417 mg/mL of Fast Red (Sigma-Aldrich)) was added for 1 hr at room temperature. Then, cells were washed with water 3 times and images were taken under the microscope.

### 2.6. Alizarin Red S Staining for Mineralized Matrix Formation

On day 21 of osteoblast differentiation, cells were washed twice with PBS and fixed with 4% paraformaldehyde for 10 min at room temperature. The fixative was rinsed, and the cells were then washed 3 times with distilled water and stained with the 2% Alizarin Red S Staining Kit (ScienCell Research Laboratories, Cat. No. 0223) for 10–20 min at room temperature. Later, the cells were washed with water and images were taken under the microscope.

### 2.7. RNA Extraction and cDNA Synthesis

Total RNA was extracted from cell pellets on day 10 of osteoblast differentiation using the Total RNA Purification Kit (Norgen Biotek Corp., Thorold, ON, Canada, https://norgenbiotek.com/) according to the manufacturer's instructions. The concentrations of total RNA extracted were then measured using NanoDrop 2000 (Thermo Fisher Scientific Life Sciences). cDNA was synthesized with 500 ng of total RNA using the High-Capacity cDNA Transcription Kit (Thermo Fisher Scientific Life Sciences) according to the manufacturer's instructions.

### 2.8. Quantitative Real Time-Polymerase Chain Reaction

Quantitative Real Time-Polymerase Chain Reaction (RT-PCR) was performed with fast SYBR Green using the Applied Biosystems ViiA™ 7 Real-Time PCR System (Thermo Fisher Scientific Life Sciences). Primers used in this study are listed in Table 1. Relative expression was calculated using the 2∆CT value method, and analysis was made as previously described [15].

### 2.9. Gene Expression Profiling by Microarray

One hundred fifty nanograms of total RNA from day 10 of osteoblast differentiation was labeled using a low-input Quick Amp Labeling Kit (Agilent Technologies, Santa Clara, CA, http://www.agilent.com) and hybridized to the Agilent Human SurePrint G3 Human GE 8 × 60 k microarray chip. All microarray experiments were performed at the Microarray Core Facility (Stem Cell Unit, Department of Anatomy, King Saud University College of Medicine, Riyadh, Saudi Arabia). Data were normalized and analyzed using GeneSpring 13.0 software (Agilent Technologies). Pathway analyses were completed using GeneSpring 13.0 as described previously [16]. Twofold cutoff and *P*(corr) < 0.05 (Benjamini-Hochberg multiple testing corrected) were set to define significantly changed transcripts. Pathway and functional annotation analyses were conducted using the Ingenuity Pathway Analysis (Ingenuity Systems, http://www.ingenuity.com/) [17, 18]. Downregulated genes ≤ 2 FC (fold change) and corrected *P* value < 0.05 were chosen for analysis. Enriched network categories were algorithmically generated based on their connectivity and ranked according to *Z* score.

### 2.10. In Vivo Ectopic Bone Formation Assay

Ethical approval for all animal experiments was obtained from the Animal Care Committee of King Saud University. In vivo experiments were performed as previously described [19]. In brief, cells were trypsinized to a single-cell suspension and resuspended in culture medium with/without the small molecule inhibitor, BMS-833923. Around 5 × 10^5^ cells were seeded onto 40 mg Triosite hydroxyapatite-tricalcium phosphate granules per implant (HA/TCP, Biomatlante/Zimmer, Albertslund, Denmark) with 0.5 to 1 mm granules, and kept overnight at 37°C and 5% CO_2_. HA/TCP granules in combination with cells were then implanted subcutaneously (four implants per cell line) in the dorsolateral area of immune-compromised nude mice for 8 weeks. The implants were recovered, fixed in formalin, decalcified using formic acid solution (0.4 M formic acid and 0.5 M sodium formate) for three days, embedded, and sectioned at 4 *μ*m. Staining was performed with hematoxylin and eosin or Sirius Red to identify areas of formed bone.

### 2.11. Quantification of Ectopic Bone Formation

Slides were digitized using a high-resolution whole-slide digital ScanScope scanner (Aperio Technologies, Inc.). The digital images from hematoxylin and eosin-stained slides were viewed and quantified using the tools of ImageJ software. The whole implant was contoured to acquire the total implant area in pixels (TA). All areas of bone are selected to obtain total bone area in pixels (BA). The BA/TA ratio was calculated and reported as percentage (*n* = 3 sections per implant and 4 implants/condition). The digital images from Sirius red-stained slides were viewed and analyzed using Aperio's viewing and image analysis tools. In each slide, five rectangular fields with a fixed area of 1.18 mm^2^ (total analysis area) were randomly selected. A color deconvolution algorithm (Aperio Technologies, Inc.) was employed to measure areas of the red color of stained collagen (positive staining of Sirius red), and its percentage relative to the total area was calculated (*n* = 4 implants per treatment).

### 2.12. Statistical Analysis

Statistical analysis and graphing were assessed using Microsoft Excel 2010 and GraphPad Prism 6 Software (GraphPad Software, San Diego, CA, U.S.A.), respectively. Results were presented as mean ± SEM from at least two independent experiments. An unpaired, two-tailed Student's *t*-test was used to calculate statistical significance, and *P* values < 0.05 were considered statistically significant.

## 3. Results

### 3.1. BMS-833923 Inhibited Osteoblast Differentiation of hMSCs

**Figure 1:**
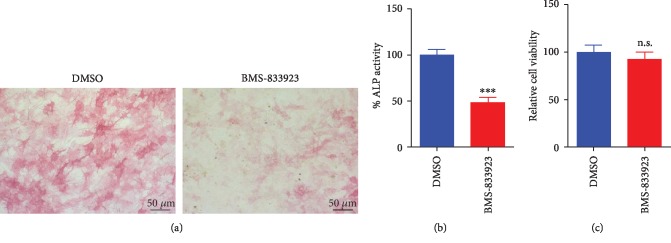
Effects of BMS-833923 treatment on the osteoblast differentiation of human bone marrow skeletal (mesenchymal) stem cells (hMSCs). (a) Representative alkaline phosphatase (ALP) staining of BMS-833923-treated hMSCs (3.0 *μ*M) on day 10 postosteoblastic differentiation compared to DMSO-treated control cells. Magnification: 10x. (b) Quantification of ALP activity in BMS-833923-treated hMSCs (3.0 *μ*M) on day 10 postosteoblastic differentiation compared to DMSO-treated control cells. (c) Assay for cell viability using alamarBlue assay BMS-833923-treated hMSCs (3.0 *μ*M) on day 10 postosteoblastic differentiation compared to DMSO-treated control cells. Data are presented as mean ± SEM (*n* = 16). DMSO: dimethyl sulfoxide.

**Figure 2:**
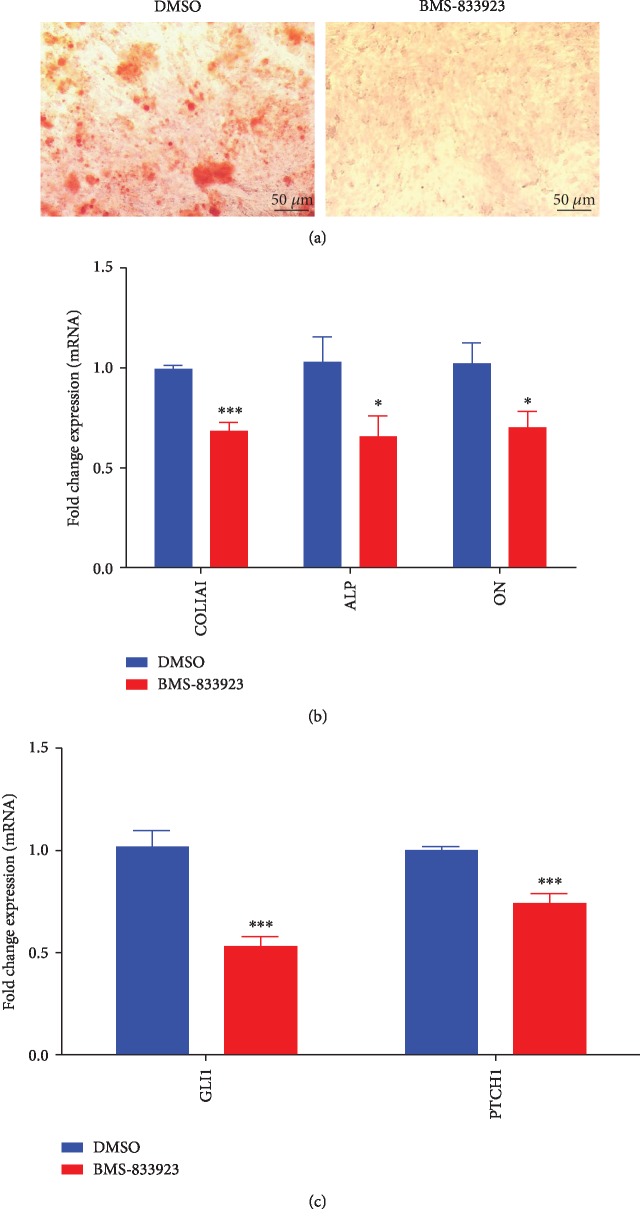
Effects of BMS-833923 treatment on human bone marrow skeletal (mesenchymal) stem cell (hMSC) functions in vitro. hMSCs were induced to osteoblast differentiation in the presence of BMS-833923 (3.0 *μ*M) or vehicle (DMSO), and osteoblast differentiation was determined by cytochemical staining with Alizarin red for an in vitro formed mineralized matrix (a) and expression of osteoblast-specific genes by quantitative RT-PCR (b). Magnification: 10x. Data are presented as mean ± SEM, *n* = 6. ^∗^*P* < 0.05; ^∗∗∗^*P* < 0.0005. (c) Expression of GLI1 and PCTH1 in hMSCs treated with BMS-833923 (3.0 *μ*M) for 24 hours and measured using qRT-PCR, *n* = 6. Abbreviations: ALP—alkaline phosphatase; COL1A1—collagen Type I Alpha 1; ON—osteonectin; DMSO—dimethyl sulfoxide; GLI1—GLI Family Zinc Finger 1; PTCH1—patched 1.

BMS-833923 was initially identified as a potent inhibitor (at 3 *μ*M) of hMSC osteoblastic differentiation based on a functional small molecule library screen [11]. hMSCs exposed to BMS-833923 (3 *μ*M) exhibited a significant decrease in ALP cytochemical staining and ALP activity compared to DMSO-vehicle control cells (Figures 1(a)–1(b)) but did not exert significant effects on hMSC cell viability (Figure 1(c)). Also, hMSCs exposed to BMS-833923 exhibited a significant reduction in mineralized matrix formation as stained with Alizarin red, compared to vehicle-treated controls (Figure 2(a)) which was associated with a significant reduction in the expression of a number of osteoblast gene markers: ALP, COL1A1, and ON (Figure 2(b)). To confirm that BMS-833923 is indeed targeting the hedgehog signaling pathway, hMSCs were treated with BMS-833923 at the same concentration (3 *μ*M), and 48 hrs later, the expression of GLI Family Zinc Finger 1 (GLI1) and Patched 1 (PTCH1), downstream readouts of the hedgehog signaling pathway, was assessed using qRT-PCR. Data presented in Figure 2(c) demonstrated significant inhibition of the hedgehog signaling pathway by BMS-833923. These data demonstrate that BMS-833923 exerts inhibitory effects on osteoblast differentiation and mature osteoblastic cell functions, through the inhibition of the hedgehog signaling pathway.

### 3.2. Global Gene Expression Identified Multiple Altered Signaling Pathways in BMS-833923-Treated hMSCs

**Figure 3:**
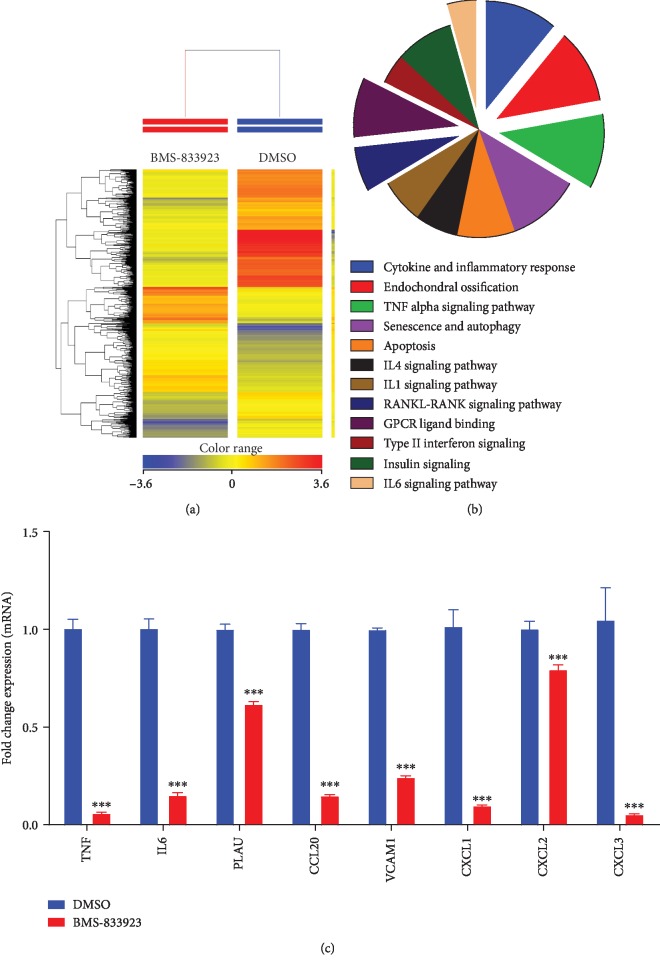
BMS-833923 exerts significant effects on multiple signaling pathways during osteoblast differentiation of human bone marrow skeletal (mesenchymal) stem cells (hMSCs). hMSCs were induced to osteoblast differentiation in the presence of BMS-833923 (3.0 *μ*M) or vehicle (DMSO). (a) Heat map and unsupervised hierarchical clustering performed on differentially expressed genes during osteoblastic differentiation. (b) Pie chart illustrating the distribution of selected intracellular signaling pathways enriched in the downregulated genes identified in BMS-833923-treated hMSCs compared to DMSO-treated control cells. (c) Validation of a selected panel of downregulated genes in BMS-833923-treated hMSCs compared to DMSO-treated control using qRT-PCR. Gene expression was normalized to *β*-actin. Data are presented as mean fold change ± SEM (*n* = 6), ^∗∗∗^*P* < 0.0001.

To understand the molecular mechanism by which BMS-833923 reduces osteoblastic differentiation, we performed global gene expression profiling in BMS-833923-treated hMSCs compared to vehicle-treated controls. Heat-map clustering revealed consistent changes in gene expression in BMS-833923-treated hMSCs compared to controls (Figure 3(a)). We identified 348 upregulated and 540 downregulated genes (fold change ≥ 2.0; *P*(corr) < 0.05) (Supplementary Tables [Supplementary-material supplementary-material-1] and [Supplementary-material supplementary-material-1]). Pathway analysis of the downregulated genes identified several differentially regulated signaling pathways that are associated with osteoblastic differentiation including GPCR, RANK-RANKL, insulin, TNF alpha and IL6, and cytokine and inflammatory response signaling (Figure 3(b)). A number of genes, including TNF, IL6, PLAU, CCL20, VCAM1, CXCL1, CXCL2, and CXCL3, were selected for a further validation using qRT-PCR, which together were in concordance with the microarray data (Figure 3(c)).

**Figure 4:**
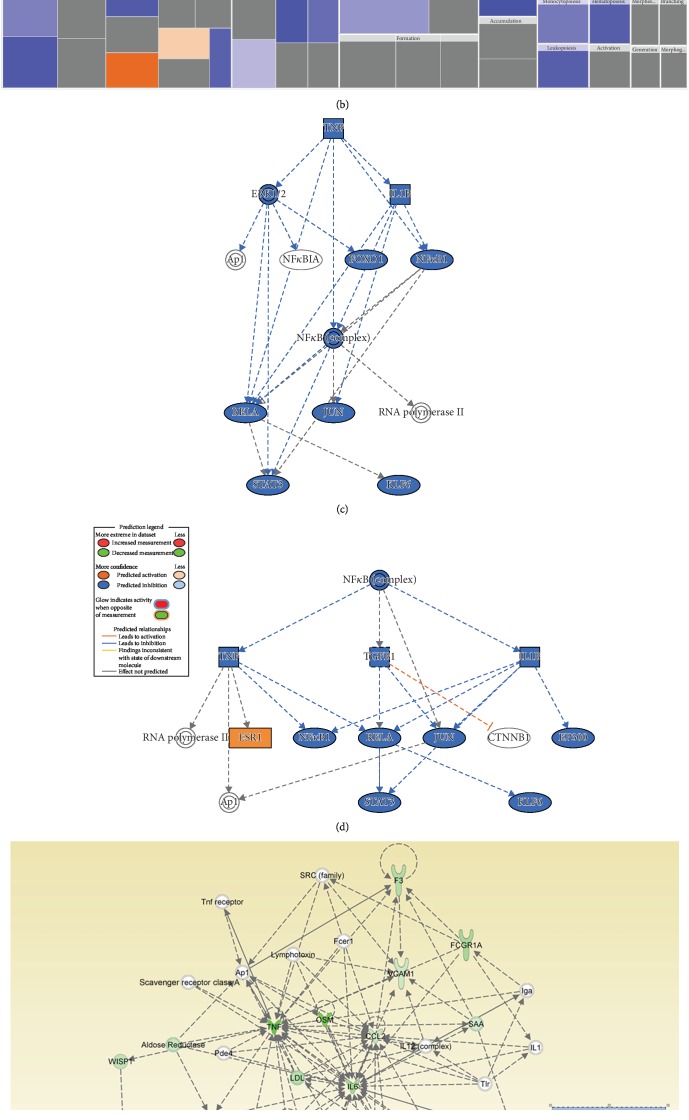
Bioinformatic analysis of signaling networks regulated in BMS-833923-treated human bone marrow skeletal (mesenchymal) stem cells (hMSCs). (a) Disease and function heat map depicting activation (red) or inhibition (blue) of the indicated functional and disease categories identified in the downregulated transcripts in BMS-833923-treated hMSCs. (b) Heat map illustrating the tissue development functional category. (c) Illustration of the TNF and (d) NF*κ*B networks with predicted activated state based on transcriptome data. Figure legend illustrates the interaction between molecules within the network. (e) Illustration of the connective tissue disorders, organismal injury and abnormalities, and skeletal and muscular disorder network.

To determine the possible mechanisms of action, we determined the functional categories and signaling networks regulated by BMS-833923 during osteoblastic differentiation of hMSCs. The identified downregulated genes were subjected to core significance analysis using a manually curated human functional annotation and network database (Ingenuity Pathway Analysis). This analysis revealed a significant reduction in gene expression in several functional gene categories including those involved in tissue development, inflammatory responses, and connective tissue diseases (Figure 4(a)). The tissue development functional category is illustrated in Figure 4(b). Follow-up upstream regulator analysis characterized a number of suppressed networks including TNF and NF*κ*B and subsequent suppression of STAT signaling (Figures 4(c) and 4(d)). The Connective Tissue Disorder, Organismal Injury and Abnormality, and Skeletal and Muscular Disorder networks were among the top identified networks that are regulated in response to BMS-833923 treatment of hMSCs (Figure 4(e)). Our data suggest that BMS-833923 plays a role in regulating the function of a number of signaling networks that are associated with the inhibition of osteoblastic differentiation of hMSCs.

### 3.3. BMS-833923 Reduced In Vivo Ectopic Bone Formation

**Figure 5:**
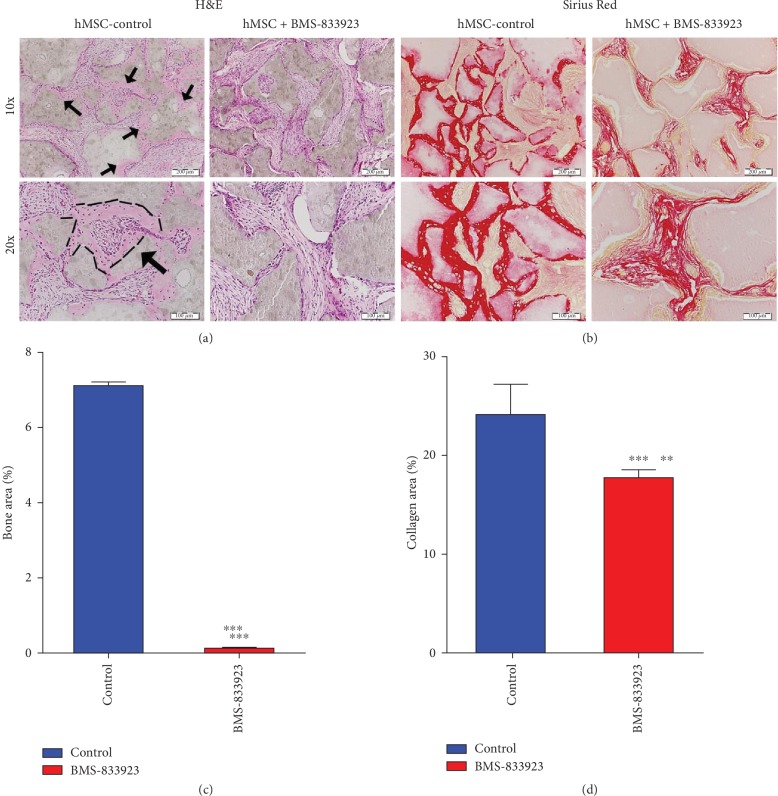
BMS-833923 reduces in vivo ectopic bone formation. BMS-833923-treated human bone marrow skeletal (mesenchymal) stem cells (hMSCs) and vehicle-treated control cells were implanted with hydroxyl apatite/tricalcium phosphate (HA/TCP) subcutaneously into immune deficient mice. The histology of in vivo bone formation was examined in H&E- (a) and Sirius red- (b) stained sections. Black arrows in (a) refer to formed bone. In Sirius red-stained slides (b), red color identifies collagen tissue staining. Magnification: 10x (first row; scale bar = 200 *μ*m) and 20x (second row; scale bar = 100 *μ*m). *n* = 3 implants/treatment. ^∗∗^*P* < 0.001; ^∗∗∗^*P* < 0.0001. H&E: hematoxylin and eosin.

To further study the role of BMS-833923 in regulating osteoblast differentiation in vivo, we determined the amount of bone formed in vivo by hMSCs treated with BMS-833923 or DMSO vehicle-treated control, following subcutaneous implantation into immune deficient mice. BMS-833923-treated hMSCs exhibited a significant reduction in ectopic bone formation capacity (Figures 5(a)–5(b)). Quantitative histological analysis revealed 90% (*n* = 3, *P* < 0.0001) reduction of bone area (Figure 5(c)) and 30% reduction in collagen matrix formation (*n* = 3, *P* < 0.001) (Figure 5(d)).

## 4. Discussion

MSCs are multipotent stem cells that reside in the bone marrow and have the potential to differentiate into various mesoderm-type cells including bone-forming osteoblasts [5]. The molecular processes and intracellular signaling pathways regulating osteoblastic differentiation are under investigation with the aim of better understanding the pathogenesis of bone disorders and identifying novel approaches for therapies [6, 20].

Chemical biology approaches employing small molecule inhibitors targeting specific intracellular signaling pathways have been widely employed to dissect molecular mechanisms regulating stem cell fate due to their known mechanism of action and biological potency that make them suitable for use in both in vitro and in vivo studies [11, 12]. In the current study, we identified BMS-833923, a small molecule inhibitor of the Hh signaling pathway, through a small molecule library screen, as a powerful inhibitor of osteoblast differentiation of hMSCs in vitro and in vivo.

BMS-833923 is a small-molecule Smoothened (SMO) inhibitor, and thus it inhibits the sonic hedgehog (SHH) signaling. In the absence of Hh, a cell-surface transmembrane protein Patched (PTCH) prevents the high expression and activity of SMO. When extracellular Hh is present, it binds to and inhibits Patched, allowing SMO to accumulate and transmit intracellular signaling [2, 21, 22]. BMS-833923 has been as an experimental oral drug in advanced cancer patients due to its antiproliferative effects. Here, we demonstrate that BMS-833923 treatment can regulate stem cell functions and that it can reduce osteoblast differentiation both in vitro and in vivo.

Global gene expression profiling of hMSCs treated with BMS-833923 identified significant changes in several intracellular signaling pathways including GPCR [23], pathways involved in endochondral ossification [24], RANK-RANKL [25], insulin [26], TNF alpha [27], IL6 [28], and cytokine and inflammatory response signaling [29] known to regulate osteoblast differentiation and function. These data suggest that hH signaling is not only important for embryonic bone development but may also play a role in the regulation of adult bone cells. During early stages of development, Shh regulates the patterning of the future limb [30–32], while Ihh acts later in the process of endochondral bone formation in limb development. Together with parathyroid hormone-related peptide, Ihh controls the growth plate and long bone development [33–35]. Hh signaling also plays a crucial role in dermal bone formation and intramembranous ossification by regulating osteogenesis rather than proliferation [36]. In adults, Hh signaling is required for bone homeostasis [37]. Shh regulates osteoblast proliferation and differentiation and osteoclast formation at the remodeling site of fractures [38]. Notably, we did not observe direct changes in HH genes based on the microarray data; however, we observed the suppression of the GPCR, which is downstream of the HH signaling pathway (Figure 3(b)). This could be explained by the fact that the microarray experiment was conducted on day 10 postdifferentiation; therefore, at that time point, and since hMSCs have already differentiated, we anticipate that the HH pathway is no longer active in those cells. It is plausible that BMS-833923 exerts its inhibitory effects at an early stage during hMSC differentiation (Figure 2(c), at 48 hrs). On day 10, the most significant changes were in processes related to osteogenesis and cytokine production.

BMS-833923-treated hMSCs exhibited significant downregulation in members of the two major chemokine subfamilies—CC and CXC, i.e., CXCL1, CXCL2, CXCL3, CXCL8, CCL2, CCL20, CCL4L2, and CCL21 (Figure 3(c) and Supplementary [Supplementary-material supplementary-material-1]). These chemokines are known to play a role during bone repair through the recruitment of progenitor cells to bone regeneration sites [39, 40]. In addition, we identified NF*κ*B and STAT signaling among the top inhibited signaling pathways in BMS-833923-treated hMSCs; both are known to play a role in osteoblast differentiation and bone formation.

BMS-833923 has been suggested to be relevant for the therapy of a number of cancer types due to its efficacy in inhibiting hH signaling. BMS-833923 inhibited the growth of tumor cell lines derived from patients with hematological malignancies such as CML, ALL, and AML [41] and it has also in vivo inhibitory effects on tumor growth of medulloblastoma and pancreatic carcinoma in animal models after a single oral dose [21]. Finally, some studies have suggested that BMS-833923 can be used in the management of cancer bone diseases. In vitro treatment with BMS-833923 inhibited the growth of myeloma cells and decreased the percentage of ALDH+ cancer stem cells in the bone marrow of patients with multiple myeloma [42, 43].

To date, several clinical trials tested the safety and efficacy of BMS-833923 in various human cancers (https://clinicaltrials.gov/); however, the effect of BMS-833923 on stem cell differentiation has not been addressed thus far. Currently, there are several FDA-approved sonic hedgehog inhibitors for the treatment of patients with various cancers. The majority of sonic hedgehog inhibitors target SMO (i.e., Vismodegib and Sonidegib), while other inhibitors target GLI1 (Arsenic Trioxide and Genistein) or SHH itself (5E1 and Robotnikinin) [44]. BMS-833923 also targets SMO; however, it remains to be addressed if various HH pathway inhibitors exert similar effects on hMSC differentiation.

## 5. Conclusions

Our results demonstrate the efficient inhibition of osteoblast function in vitro and in vivo suggesting that BMS-833923 can be employed in the treatment of bone diseases with enhanced bone formation, e.g., sclerotic bone metastases.

## Figures and Tables

**Table 1:** List of SYBR Green primers used in current study.

Gene name	Forward primer	Reverse primer
ACTB	5′AGCCATGTACGTTGCTA3′	5′AGTCCGCCTAGAAGCA3′
ALPL	5′GGAACTCCTGACCCTTGACC3′	5′TCCTGTTCAGCTCGTACTGC3′
COL1A1	5′GAGTGCTGTCCCGTCTGC3′	5′TTTCTTGGTCGGTGGGTG3′
ON	5′GAGGAAACCGAAGAGGAGG3′	5′GGGGTGTTGTTCTCATCCAG3′
LIF	5′GCCACCCATGTCACAACAAC3′	5′CCCCCTGGGCTGTGTAATAG3′
CXCL1	5′CCAGCTCTTCCGCTCCTC3′	5′CACGGACGCTCCTGCTG3′
CXCL2	5′GGGGTTCGCCGTTCTCGGA3′	5′TGCGAGGAGGAGAGCTGGCAA3′
CXCL3	5′CGCCCAAACCGAAGTCATAGCCA3′	5′TGGTAAGGGCAGGGACCACCC3′
IL6	5′CGAGCCCACCGGGAACGAAA3′	5′GGACCGAAGGCGTTGTGGAG3′
PLAU	5′ACTCCAAAGGCAGCAATGAAC3′	5′GTGCTGCCCTCCGAATTTCT3′
TNF	5′ACTTTGGAGTGATCGGCC3′	5′GCTTGAGGGTTTGCTACAAC3′
CCL20	5′CGAATCAGAAGCAGCAAGCAA3′	5′TTGCGCACACAGACAACTTT3′
VCAM	5′TGTTTGCAGCTTCTCAAGCTTTT3′	5′GATGTGGTCCCCTCATTCGT3′
GLI1	5′CTGGATCGGATAGGTGGTCT3′	5′CAGAGGTTGGGAGGTAAGGA3′
PTCH1	5′TGACCTAGTCAGGCTGGAAG3′	5′GAAGGAGATTATCCCCCTGA3′

## Data Availability

Additional data are available as supplementary data.

## Supplementary Materials

Supplementary Materials Supplementary Table 1: upregulated genes in hBMSCs treated with BMS-833923 compared to DMSO on day 10 of osteoblastic differentiation. Supplementary Table 2: downregulated genes in hBMSCs treated with BMS-833923 compared to DMSO on day 10 of osteoblastic differentiation.
